# Substance use disorders, trauma, and PTSD

**DOI:** 10.3402/ejpt.v6.27632

**Published:** 2015-04-08

**Authors:** Wim Van den Brink

**Affiliations:** Department of Psychiatrie, Academic Medical Center, University of Amsterdam, Amsterdam, The Netherlands

Substance use disorders (SUDs) and traumas frequently co-occur. A recent German study found that 66% of the women with a diagnosis of opioid dependence also reported sexual abuse, whereas the figure for men was 11% (Schäfer et al., 2014). In a similar study among alcohol-dependent patients, 35% of the female patients and 6% of male patients were sexually abused as a child (Schäfer et al., 2009). In one of our own studies among treatment-seeking alcohol-dependent patients (80% male), we found the following figures: 24% sexually abused as a child, 15% physically abused as a child, 16% witnessed domestic violence as a child, 42% physically abused as an adult, and 11% sexually abused as an adult (Langeland, Draijer, & Van den Brink, 2002). Therefore, it is hardly surprising that nearly 20% of these patients were diagnosed with a DSM-III-R posttraumatic stress disorder (PTSD). This lecture will focus on the latter group of patients: SUD patients with PTSD.

In the general population, approximately 10% of women and 5% of men meet criteria for a lifetime diagnosis of PTSD. However, for patients with alcohol dependence, the figures are approximately 25% (OR 3.6) and 10% (OR 3.2), respectively. Conversely, about 35% of people diagnosed with PTSD also have an alcohol use disorder (Kessler, Sonnega, Bromet, Hughes, & Nelson, 1995).

Among treatment-seeking SUD patients, 20–50% have a lifetime diagnosis of PTSD, whereas 15–40% met criteria for PTSD in the last year (Brady, Killeen, Brewerton, & Lucerini, 2000; Van Dam, Ehring, Vedel, & Emmelkamp, 2010). Conversely, about 20% of the treatment-seeking patients with PTSD also meet criteria for a current SUD. This is important because this comorbidity is associated with more severe PTSD and more severe SUD and with negative treatment outcomes for both PTSD and SUD (Brady et al., 2000). Furthermore, episodes of “re-experiencing” are associated with an increase in substance abuse and an increased risk of relapse, whereas withdrawal and abstinence are associated with an increase of PTSD symptoms (Brown, Stout, & Gannon-Rowley, 1998).

There are multiple theories about the reasons for the high co-occurrence of SUD and PTSD. Some believe that PTSD precedes SUD and that SUD is the consequence of attempts to self-medicate PTSD symptoms. Others believe that SUD occurs first with substance-use-related traumas as an adult resulting in comorbid PTSD. Finally, some consider that both SUD and PTSD are the result of a shared vulnerability due to some genetic predisposition or early childhood trauma. Research shows that PTSD precedes SUD far more frequently than the other way around, that PTSD and SUD are both very often preceded by trauma in early childhood, and that there is some genetic overlap for the two disorders (Kessler et al., 1995; Sartor et al., 2011). On the basis of these etiological considerations, it seems that the treatment of PTSD (as a causal factor in the development of SUD) is more important than the treatment of SUD in patients with both disorders. However, in the course of the disorder, the course of SUD may become rather independent and simultaneous treatment of both disorders would be most preferable. However, this still raises the question of whether treatment should be (predominantly) psychotherapeutic or pharmacological and, furthermore, what kind of psychotherapy or medication.

A large number of studies have looked at the effect of psychotherapeutic treatments of SUD patients with comorbid PTSD. The results of these studies have been summarised in two recent reviews (Najavits & Hien, 2013; Van Dam, Vedel, Ehring, & Emmelkamp, 2012). Both reviews deal with cognitive behavioural therapy aimed at stabilisation without exposure (such as Seeking Safety) as well as cognitive behavioural therapy with exposure. The latter review contains 35 studies and pays a lot of attention to Seeking Safety (22 studies), a treatment developed by Najavits. The review strongly recommends Seeking Safety as the treatment of choice. The review by Van Dam et al. contains 17 studies and is quite critical about the results of both forms of cognitive behavioural therapy. However, in contrast to Najavits and Hien, it cautiously recommends treatments with exposure, arguing that this form of treatment shows the best results with non-addicts and that it is also more appreciated by comorbid patients. Both reviews admit that none of the psychotherapeutic interventions for PTSD had a clear effect on substance use.

There are various different effective medications available for the treatment of nicotine, heroine, alcohol, and cannabis dependence, and there are some promising medications for the treatment of cocaine and other stimulant use disorders (Van den Brink, 2012). However, the number of studies about the pharmacological treatment of PTSD in patients with a comorbid SUD is rather limited, and the methodological quality of many of these studies is low. Nevertheless, a recent review (Norman et al., 2012) considers the following medications: the antidepressant sertraline, the antipsychotic quetiapine, and the anti-epileptic topiramate. Sertraline seems to work best in comorbid patients with a primary diagnosis of PTSD. However, sertraline might be contraindicated in a primary alcohol-dependent patient with secondary PTSD because in these patients sertraline may lead to increased alcohol use (Brady et al., 2005). The advantage of topiramate is that alcohol detoxification can be initiated at the beginning of the treatment because it prevents withdrawal symptoms and complications as well as reducing substance use and PTSD symptoms.

An important problem that needs more attention is that most interventions have no effect on nightmares or other sleeping problems associated with either PTSD or SUD. A recent review (Nappi, Drummond, & Hall, 2012) concludes that the best psychotherapeutic treatment for nightmares in patients with PTSD is a cognitive behavioural intervention with exposure. Also, one or more of the following medications may be effective: topiramate, prazosin, quetiapine, duloxetine, and gabapentin. It should be noted that topiramate and prazosin are probably also effective in the treatment of alcohol dependence (Simpson et al., 2009; Van den Brink, 2012).

Based on this overview of the literature, the author recommends: 1) all treatment-seeking SUD patients should been screened for PTSD; 2) all treatment-seeking PTSD patients should be questioned about their alcohol and drug use; 3) all patients with PTSD and/or SUD should be screened for the presence of early childhood trauma; 4) the presence childhood trauma should always be validated and possibly treated; 5) the relationship between PTSD symptoms and substance use should be assessed and monitored; 6) the patient's preference for abstinence or controlled use should be taken into account because both drug use and withdrawal can worsen the course of PTSD symptoms; 7) all patients with PTSD and SUD should be screened for sleeping disorders; 8) it is preferable to treat PTSD and SUD simultaneously either with one or two different therapists; 9) cognitive behavioural therapy with exposure is preferred (including eye movement desensitisation and reprocessing), possibly in combination with pharmacotherapy for addiction and/or PTSD; 10) there is no role for benzodiazepines or tricyclic antidepressants in the treatment of patients with SUD and PTSD; and 11) there might be a role for topiramate, prazosin, or gabapentin in the treatment of nightmares in patients with PTSD and SUD.
